# Physical activity habits and their effects on quality of life in patients with addiction: data from the Czech Republic

**DOI:** 10.1007/s12144-023-04402-w

**Published:** 2023-03-28

**Authors:** Michaela Zahrádka Köhlerová, Zdeňka Fišerová, Marek Páv

**Affiliations:** 1 Psychiatric Hospital Bohnice, Centre for Psychosomatic Therapy and Rehabilitation, PN Bohnice, Ústavní 91, 181 02 Prague, Czech Republic; 2 Department of Addictology, First Faculty of Medicine, Charles University, General University Hospital in Prague, Kateřinská 1660/32, 121 08 Prague 2, Czech Republic; 3 Third Faculty of Medicine, Charles University, Ruská 87, 100 00 Prague 10, Czech Republic; 4 Department of Psychiatry, First Faculty of Medicine, Charles University in Prague, General University Hospital in Prague, Kateřinská 1660/32, 121 08 Prague 2, Czech Republic

**Keywords:** Substance use disorders, Addiction, Physical activity, SF-36, Quality of life

## Abstract

Addiction, including substance use disorder (SUD), is a complex condition that can cause serious health problems and negatively affect patient quality of life. Physical activity is known to improve the physical and mental health of patients with SUD. This study aims to determine the relationship between regular physical activity (RPA) and quality of life in SUD patients enrolled in inpatient SUD treatment (n = 159). We divided patients into four groups based on their RPA before and during hospitalization. The SF-36 self-report questionnaire was used to assess quality of life. We found that SUD patients had worse quality of life than a representative sample of the Czech population. Furthermore, we demonstrated that RPA before and during hospitalization and changes during hospitalization affect the perception of quality of life of patients with SUDs. Additionally, physically active patients showed significantly better quality of life than inactive patients. However, patients who initiated RPA during hospitalization reported worse quality of life than those who did not; in addition, this group of patients reported the worst quality of life across the most monitored parameters. We suggest that these patients represent the most vulnerable group. Changes in physical activity habits could be considered an indicator for a more intensive therapeutic focus.

**Supplementary Information:**

The online version contains supplementary material available at 10.1007/s12144-023-04402-w.

## Introduction

The topic of substance use disorders (SUDs) is of continuous interest in Europe, including the Czech Republic, where significant numbers of patients with addiction are present among the population (Formánek et al., 2019). Indeed, SUD is regularly associated with social and economic problems and shortens life while worsening its quality (Vancampfort et al., 2018). It is well-known that stress increases vulnerability to addiction and risk of relapse. Recently, one of the main sources of stress has been COVID-19. Therefore, the impact of COVID-19 and related lockdowns on lifestyle habits, psychological well-being and quality of life is being studied (Odone et al., 2020).

Patients with SUDs report decreased quality of life (QoL) (Hoseinifar et al., 2011; Pasareanu et al., 2015; Smith & Larson, 2003), regardless of the abused substance (Denis et al., 2012). More than 30% of patients experience chronic health problems due to substance abuse, such as liver disease, tuberculosis (TBC), HIV/AIDS, cancer, hepatitis C, and diabetes (Eddie et al., 2019). Additionally, decreased social well-being and mental anxiety have been reported (Muller et al., 2016).

Addiction treatment in the Czech Republic has a tradition starting in the 1950s and has developed inpatient treatment for people with SUDs utilizing a comprehensive therapeutic program (Mravcik et al., 2011) that has incorporated continuous improvements through the present day. Treatment includes 5 to 12 days of initial detoxification and subsequent treatment consists mainly of group psychotherapy, minimal medication treatment, hospital treatment in a community ward, social consulting, and physical activity (PA) (Kalina, 2007).

Many findings have shown that regular PA (RPA) increases physical and mental health in patients with addiction (Brown et al., 2016; Kim, 2013; Malt, 2008; Peluso & Guerra de Andrade, 2005; Rawson et al., 2015; Trivedi et al., 2017). PA decreases craving (Roessler et al., 2017) and thus significantly contributes to the long-term maintenance of abstinence (Brown et al., 2009). Furthermore, PA improves psychosocial functioning in patients (Aarde et al., 2015), generally helps to improve QoL (Warburton et al., 2006) and could even reduce mortality, as shown in a recent study in major depression (Murri et al., 2019). Exercise is an accessible and affordable complement to other forms of treatment (Read & Brown, 2003). Additionally, using various treatment services and modalities has positive effects on treatment outcomes (Abbott et al., 1998; McLellan et al., 1994). Patients also benefit from sustained PA, as several studies have suggested positive effects of controlled PA on abstinence after discharge from the hospital (Rawson et al., 2015; Sinyor et al., 1982; Trivedi et al., 2017).

PA programs for patients with addiction in Psychiatric Hospital Bohnice (PHB) in Prague, Czech Republic, include (among others) group exercises, yoga, jogging, bodybuilding, and relaxation. However, outcomes and benefits of PA for patients have not been systematically monitored, although this monitoring is essential, as adopting healthy lifestyle habits is highly desirable during treatment as an alternative to addictive behavior and should constitute a fundamental treatment goal (Colledge et al., 2018). Furthermore, regular participation in PA helps with reintegration into everyday life (Kuda & Kalina, 2008), allowing patients to recognize their own needs and tailor a PA program to address those needs (Muller et al., 2016).

Monitoring patient QoL in the treatment of addiction presents a diagnostic tool (Rudolf & Watts, 2002) that helps evaluate the patient’s clinical status or predict an upcoming relapse (Srivastava et al., 2009). Therefore, interventions increasing QoL should be preferred (Muller et al., 2016).

Although PA is known to improve QoL of people, including patients with SUD, it has not yet been studied in the Czech Republic. Therefore, this study aimed to evaluate patient QoL based on their PA habits.

## Materials and Methods

### Participants, data collection and analysis

We included a total of 159 patients in this study. The group consisted of patients with mental and behavioral disorders due to the use of alcohol (F10) (60/37.7%), mental and behavioral disorders due to the use of sedatives or hypnotics (F13) (6/3.8%), mental and behavioral disorders due to multiple drug use and the use of other psychoactive substances (66/41.5%), gambling disorder (4/2.5%) and dual diagnosis (23/14.5%). All patients initially underwent standardized therapy procedures for 12 weeks, which focused on psychotherapeutic interventions and routine day regimes in a community of other patients. The 1st to 3rd week of hospitalization consisted of detoxification and support for the physical and psychological stabilization of the patients, which is an indispensable condition for subsequent demanding therapy. We applied exclusion criteria, which included receiving chemotherapy treatment or having an active oncological disorder, an acute cardiometabolic or lung condition, a transmissible infectious disease, or an unstable severe mental illness.

Data were collected in PHB in Prague, Czech Republic, in four dedicated wards providing care to alcohol- or drug-addicted patients and addicted patients with other psychiatric diseases according to the International Statistical Classification of Diseases and Related Health Problems (ICD-10). We surveyed each participant once during the 4th to 12th week of hospitalization; we collected 159 fully answered questionnaires out of 167 surveyed patients.

An ethical committee approved the survey of PHB, and all patients were informed about the use of their answers in the study according to Czech and international legal and ethical standards (acknowledged by signed informed consent).

### Questionnaires

We monitored QoL of patients with SUDs with the Czech language version of the 36-Item Short-Form Health Survey (SF-36) questionnaire (Sobotík, 1998). The SF-36 is a standard tool for evaluating the Health-Related Quality of Life index (HRQL) for many somatic diseases, including diabetes or cardiovascular diseases (Sobotík, 1998), and psychiatric disorders or for monitoring significant life changes such as menopause (Moravcova & Mares, 2011). The SF-36 consists of 36 questions divided into eight domains: physical functioning, role limitation-physical, social functioning, role limitation-emotional, pain, mental health, vitality, and general health (Ware & Sherbourne, 1992). Regarding the study being localized in the Czech Republic, we compared current results to answers to the SF-36 questionnaire from previous studies in the local population (Bártlová et al., 2020). This normative group consisted of 1992 individuals that provided a representative sample of Czech citizens aged 40 and older (Bártlová et al., 2020).

All responses were collected in standard paper format (completed by patients). We administered the sociodemographic data questionnaire to each patient. To obtain PA information, the patients were asked to answer two questions regarding their habits and preferences in relation to PA before and during hospitalization: 1) “Did you practice regular physical activity (RPA) before hospitalization?” and 2)” Do you practice RPA during hospitalization (in addition to the official PA programs in the hospital)?”. If they answered “yes”, they were subsequently asked to provide specific information related to their answer and indicate the length and frequency of the exercise. RPA was defined as any PA regularly engaged in for at least 30 min per week. A list of compulsory and voluntary interventions is shown in (Supplementary Table I).

Based on the information obtained on RPA before and during hospitalization, the study participants were divided into four groups. Group A (27.0%) consisted of patients who reported RPA before and during hospitalization. Group B (32.1%) patients reported RPA before hospitalization; however, they were not spontaneously active in the hospital. Patients of Group C (17.6%) were physically inactive before hospitalization, yet they initiated spontaneous RPA in the hospital. Group D (23.3%) patients were inactive before and after hospitalization. QoL between physically active and inactive patients before (Groups A + B vs. C + D) and during hospitalization (Groups A + C vs. B + D) and also between patients who changed their habits and stopped/started RPA after hospitalization and those who did not (A vs. B; C vs. D) was compared.

### Statistical analysis

We performed all statistical analyses by using GraphPad Prism 6 (GraphPad Software, San Diego, CA, USA). The Shapiro–Wilk normality test determined normality of the distribution. Comparisons of variables between the two groups were analyzed by unpaired t tests or Mann–Whitney tests. To compare data obtained from three and more groups of subjects, one-way ANOVAs or Kruskal–Wallis tests were conducted. Wilcoxon signed-rank tests were used for the comparison between the patient and normative groups. We calculated Pearson’s and Spearman’s correlation coefficients to assess the relationships between pairs of variables. P-values less than 0.05 were considered statistically significant.

## Results

The demographic characteristics of the subjects are summarized in Table 1. The group consisted of 80 men and 79 women with a mean age of 40.7 years. Patient QoL was not affected by sex, body mass index (BMI) or level of education. However, four parameters were positively associated with age: pain (p = 0.034), role limitation-physical (p = 0.004), physical functioning (p < 0.001) and general health (p = 0.014). Further details describing association between characteristics of patients and parameters of their QoL are shown in (Supplementary Table II). In addition, the patients undergoing their first hospitalization reported significantly better general health than those who were repeatedly hospitalized (p = 0.024). There were no differences in the demographic characteristics of the patients among the groups A-D (Table 1).

**Table 1:** Characteristics of patients

	All patients	Group A	Group B	Group C	Group D
Number, *n (%)*	159 (100)	43 (27.0)	51 (32.1)	28 (17.6)	37 (23.3)
Males/females, *n (%)*	80/79 (50.3/49.7)	19/24 (44.2/55.8)	33/18 (64.7/35.3)	11/17 (39.3/60.7)	17/20 (45.9/54.1)
Age, *years*	40 (32–49)	36 (29–42)	42 (35–52)	39 (32–47)	43 (29–55)
BMI, *kg/m*^*2*^	24.4 (22.1–27.0)	24.0 (21.8–27.1)	23.6 (22.2–25.8)	25.5 (22.4–27.1)	25.1 (22.2–29.0)
Education, *n (%)* Elementary/High school/University	27/106/26 (17.0/66.7/16.3)	3/28/12 (7.0/65.1/27.9)	7/34/10 (13.7/66.7/19.6)	6/20/2 (21.4/71.4/7.1)	11/24/2 (29.7/64.9/5.4)
First hospitalization, *n (%)*	69 (43.4)	21 (48.8)	24 (47.1)	10 (35.7)	14 (37.8)
RPA before/during hospitalization	-	Yes/Yes	Yes/No	No/Yes	No/No

The data are presented as median (interquartile range). BMI, body mass index; RPA, regular physical activity

Group A: Patients who engaged in RPA before and during hospitalization

Group B: Patients who engaged in RPA only before hospitalization (inactive during hospitalization)

Group C: Patients who engaged in RPA only during hospitalization (inactive before hospitalization)

Group D: Patients who were inactive before and during hospitalization

### Comparison of the group of patients with addiction and the normative group

QoL in the group of patients with SUDs compared to the sample group of Czech citizens is shown in Table 2. All parameters, with the exceptions of physical functioning (p = 0.631) and general health (p = 0.062), were significantly worse in the patients with addiction (p < 0.001).

**Table 2:** Comparison of the addicted patients? group with normative of the Czech population

	Group A (n = 43)	Group B (n = 51)	Group C (n = 28)	Group D (n = 37)	All patients (n = 159)	Normative* (n = 1992)	p-value
**Physical functioning**	95.4 ± 9.9	85.2 ± 18.5	90.7 ± 11.3	75.8 ± 26.7	86.7 ± 19.2	79.0 ± 23.6	0.6313
**Role-physical**	68.0 ± 36.3	55.4 ± 40.7	42.0 ± 42.0	65.5 ± 41.4	58.8 ± 40.7	73.1 ± 36.5	< 0.0001
**Role-emotional**	49.6 ± 44.5	35.9 ± 42.1	25.0 ± 39.2	46.0 ± 44.0	40.0 ± 43.2	78.5 ± 34.5	< 0.0001
**Vitality**	55.4 ± 20.8	48.2 ± 19.5	42.3 ± 22.1	47.3 ± 22.1	48.9 ± 21.2	57.0 ± 18.1	< 0.0001
**Mental health**	58.4 ± 18.7	49.6 ± 19.6	45.0 ± 23.8	51.0 ± 21.0	51.5 ± 20.8	71.0 ± 15.7	< 0.0001
**Social functioning**	58.8 ± 27.5	52.5 ± 27.4	40.2 ± 27.0	58.5 ± 29.2	53.4 ± 28.3	79.0 ± 22.3	< 0.0001
**Pain**	81.0 ± 23.8	59.7 ± 31.5	61.7 ± 29.2	65.3 ± 30.3	67.1 ± 29.9	76.9 ± 24.3	< 0.0001
**General health**	65.5 ± 22.8	52.8 ± 17.9	53.6 ± 20.2	53.7 ± 22.3	56.5 ± 21.2	58.1 ± 19.0	0.0618

* Normative is adopted from (Bártlová et al., 2020).

### Comparison of patient subgroups based on their regular physical activity habits

**Fig. 1:**
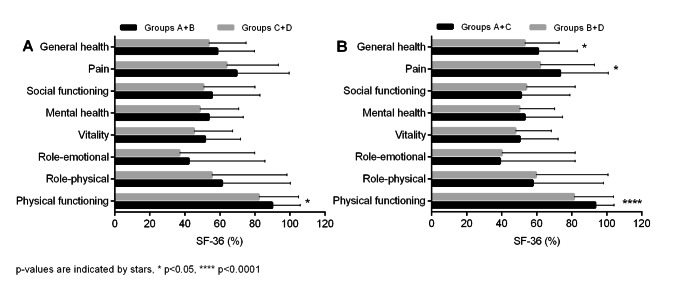
**(A)** Comparison of quality of life in the patients who reported RPA before hospitalization (Groups A and B) with physically inactive patients (Groups C and D). **(B)** Comparison of quality of life in the patients who engaged in RPA during hospitalization (Groups A and C) with that in physically inactive patients (Groups B and D). Data are presented as the mean ± standard deviation

The patients who engaged in RPA before hospitalization (Groups A and B) reported significantly better physical functioning (p = 0.025) than the inactive patients (Groups C and D). Other QoL parameters were also higher in the active patients, but these differences were not statistically significant (Fig. 1A).

The patients who engaged in RPA during hospitalization (Groups A and C) reported significantly better physical functioning (p < 0.001), pain (p = 0.027) and general health (p = 0.024) scores than those who did not (Groups B and D) (Fig. 1B).

**Fig. 2:**
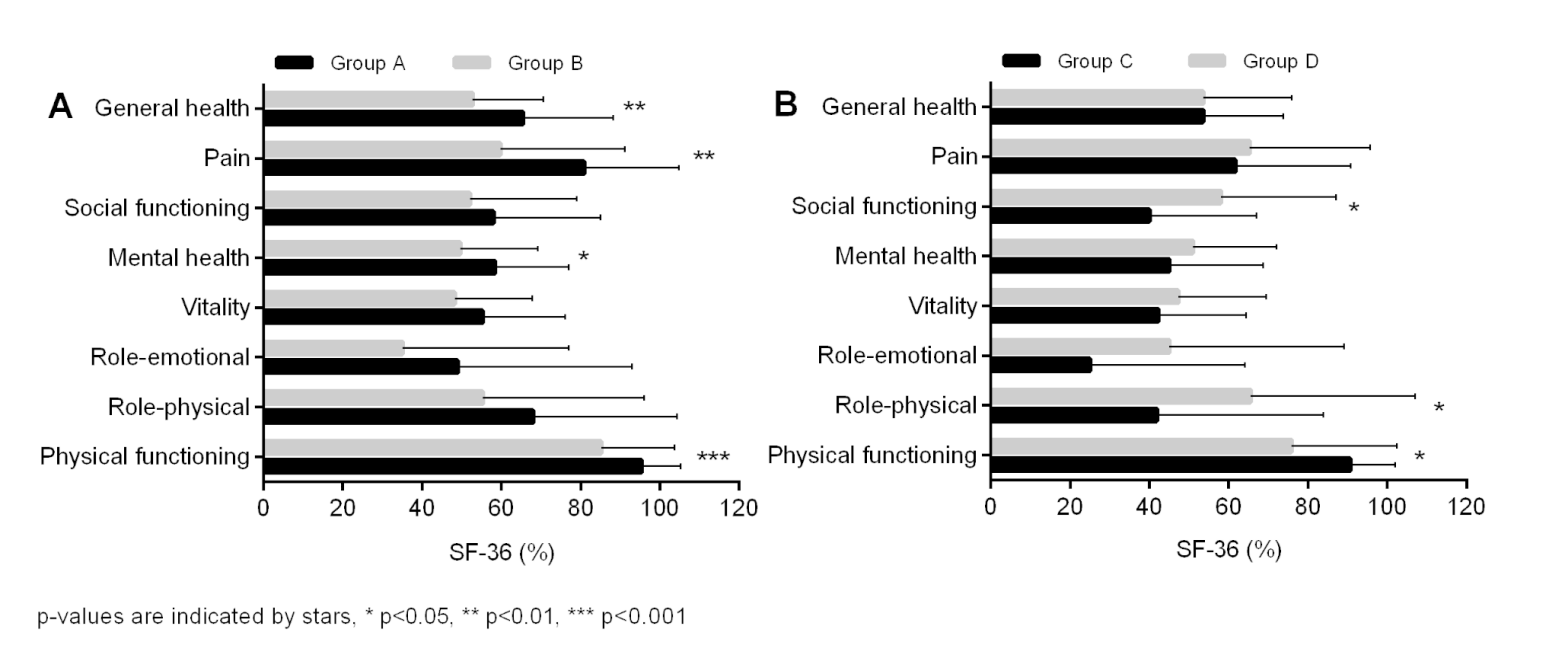
**(A)** Comparison of quality of life in the patients who engaged in RPA before and during hospitalization (Group A) with patients who stopped RPA after hospitalization (Group B). **(B)** Comparison of quality of life in the patients who did not engage in RPA before and during hospitalization (Group D) with a group who changed habits and initiated RPA after hospitalization (Group C). Data are presented as the mean ± standard deviation

The majority of patients who engaged in RPA before hospitalization changed their habits and did not engage in RPA during hospitalization (Group B). Comparison of Group B with Group A showed significantly lower QoL reported for physical functioning (p < 0.001), pain (p = 0.001), mental health (p = 0.028), and general health (p = 0.003). Other parameters were not significantly affected, although they were also decreased, compared to those in Group A (Fig. 2A).

Even though 40.9% of patients had not been active before hospitalization (Groups C and D), some (Group C) changed their habits and initiated RPA during hospitalization. In Group C, QoL, as reflected in role limitation-physical (p = 0.025) and social functioning (p = 0.015) scores, was significantly lower than that in Group D. Other parameters were also decreased in Group C, with the exception of physical functioning, which was significantly increased (p = 0.034) (Fig. 2B). Group C represented 17.6% of patients and was identified as having the worst reported QoL based on most of the monitored parameters (role limitation-physical, role limitation-emotional, vitality, mental health, and social functioning).

## Discussion

This study examined QoL of 159 patients with SUDs in relation to their PA. We found worse QoL in patients with addiction than in the representative sample from the general Czech population (Bártlová et al., 2020). We also demonstrated significantly better QoL in patients who engaged in RPA before and during hospitalization than in physically inactive patients. In addition, we found that changes in patient habits during hospitalization could affect their QoL.

A previous meta-analytic study (Wang et al., 2014) demonstrated PA to be an effective tool in SUD therapy. However, the benefits of PA on SUD treatment outcomes has not yet been fully proven (Linke & Ussher, 2015; Zschucke et al., 2012) due to a lack of standardized program methodology and program heterogeneity (Dowla et al., 2018) and inconsistent evaluation criteria (Colledge et al., 2018). However, positive effects of PA have been observed with short- or long-term exercise programs (Brown et al., 2016; Zhao et al., 2020). Although the mechanism of how PA contributes to treatment effects in the context of mental illnesses, and in particular, in the treatment of addictions, has not yet been sufficiently elucidated, many studies have been performed in other patient subgroups (Zschucke et al., 2012) or animal models (Aarde et al., 2015).

Poorer QoL of patients with addiction has been repeatedly shown (Denis et al., 2012; Hoseinifar et al., 2011; Pasareanu et al., 2015; Smith & Larson, 2003). Accordingly, our group of patients with SUDs reported significantly worse values on most parameters of the SF-36 questionnaire than the normative Czech population (Bártlová et al., 2020). Our cohort was further divided into four groups based on RPA performed before and during hospitalization to determine the relationship between RPA and QoL.

The patients who were physically active before hospitalization showed higher QoL scores on all parameters than physically inactive patients. However, statistical significance was demonstrated only for physical functioning. These findings could indicate a potential prognostic value of RPA in patients with SUD. The patients who engaged in RPA during hospitalization showed significantly better results in the SF-36 domains physical functioning, pain, and general health than those who did not. Other QoL parameters were comparable between groups. However, low rates of significance with the other parameters could be explained by multiple factors, such as a relatively low number of participants, group heterogeneity, or subjective self-reporting methods. This is consistent with data from, for example, (Sari et al., 2019) who reported no effect of an exercise intervention on QoL of patients with alcohol use disorder.

Almost 60% of the patients engaged in RPA before hospitalization. However, most of them changed their habits and stopped RPA during hospitalization. These patients showed reduced QoL on all parameters in comparison to those who continued to exercise, with the difference being statistically significant for the physical functioning, pain, mental health, and general health SF-36 domains. These results could indicate that patients would report higher QoL if they were supported and motivated not to terminate their PA during hospitalization.

Some of the patients were physically inactive before hospitalization, changed their habits and initiated RPA during hospitalization. Surprisingly, these patients reported worse QoL in all SF-36 domains, with the exception of physical functioning, than physically inactive patients and showed the worst results in role limitation-physical, role limitation-emotional, vitality, mental health, and social functioning dimensions of all groups. Reasons for reduced QoL perception in this group remain unexplained and should be targeted in future studies. However, we suggest that these patients represent the most vulnerable group. A change in their PA habits could be considered an indicator for a more intensive therapeutic focus. This is the first finding identifying such a group concerning QoL.

The results of this study should be interpreted considering some limitations. First, the study lacks a suitable sex-/age-matched control group of subjects and used a previously described group of Czech citizens as a norm. Second, the method of one-time questionnaire implementation introduces the potential for recall bias. Third, we evaluated QoL and RPA solely by self-report questionnaires; objective measurements could provide deeper insight into the relationship between PA and the therapeutic effects. Fourth, the study design was cross-sectional. Therefore, a long-term prospective study is needed to further investigate the impact of RPA on QoL.

## Conclusion

In conclusion, these data from the Czech Republic showed significantly worse QoL in SUD patients than the representative population sample. Moreover, we found that QoL in the patients with SUDs was affected by RPA performed before and during hospitalization and changes during hospitalization. In general, we demonstrated significantly better QoL in physically active patients. However, patients who initiated RPA during hospitalization showed a poorer perception of QoL than those who did not. Furthermore, this group of patients was identified as the one with the worst QoL based on most of the monitored parameters. We suggest that these patients represent the most vulnerable group and that a change in their RPA-related habits could be an indicator for a more intensive therapeutic focus.

## Electronic supplementary material

Below is the link to the electronic supplementary material.

Supplementary Material 1

Supplementary Material 2

Supplementary Material 3
