# Supplementary material for: Physical activity habits and their effects on quality of life in patients with addiction: data from the Czech Republic
Source: Curr Psychol. 2023 Mar 28:1–8. Online ahead of print. doi: 10.1007/s12144-023-04402-w (PMC10043521; doi:10.1007/s12144-023-04402-w)
Supplement: Supplementary file 1 — Supplementary Material 1 [file 12144_2023_4402_MOESM1_ESM.docx]

Supplementary Table I: Interventions

| Activity | Women |
| --- | --- |
| Compulsory activities | Daily warm-up 15 min 7 x a week, 1x weekly group exercise 30 min, 1x NW 60 min |
| Optional activity with a therapist | Nordic walking 1x a week 60 min, 1 x a week group exercise 30 min, yoga 1 x a week 60 min |
| Optional in personal leave | Individual exercise in free time 15–90 min in the gym |

| Activity | Men |
| --- | --- |
| Compulsory activities | Daily warm-up 15 min 7 x a week, 4 x a week group exercise 30 min |
| Optional activity with a therapist | 1x a week yoga 60 min, running 1x a week for 30 min, ball games with a physiotherapist 60 min, 2x a week physical activities 30 min stretching and strengthening, 1x a week 60 min yoga, |
| Optional in personal leave | Gym according to time possibilities in the holiday between intensive program 15 - 90 min, individual exercise in the holiday 15 - 90 min. |
