# Supplementary material for: Physical activity habits and their effects on quality of life in patients with addiction: data from the Czech Republic
Source: Curr Psychol. 2023 Mar 28:1–8. Online ahead of print. doi: 10.1007/s12144-023-04402-w (PMC10043521; doi:10.1007/s12144-023-04402-w)
Supplement: Supplementary file 2 — Supplementary Material 2 [file 12144_2023_4402_MOESM2_ESM.docx]

Supplementary Table II. Association between characteristics of patients and parameters of their QoL

|  | **Age** | **BMI** | **Sex**  (Males vs. Females) | **First hospitalization**  (Yes vs. No) | **Education level**  (Elementary vs. High school vs. University) |
| --- | --- | --- | --- | --- | --- |
| **Physical functioning** | p < 0.001  r = -0.308 | p = 0.307  r = -0.083 | p = 0.119 | p = 0.832 | p = 0.655 |
| **Role-physical** | p = 0.004  r = -0.227 | p = 0.855  r = -0.015 | p = 0.798 | p = 0.748 | p = 0.635 |
| **Role-emotional** | p = 0.880  r = -0.012 | p = 0.625  r = 0.040 | p = 0.365 | p = 0.560 | p = 0.310 |
| **Vitality** | p = 0.587  r = -0.044 | p = 0.977  r = -0.002 | p = 0.227 | p = 0.738 | p = 0.854 |
| **Mental health** | p = 0.541  r = 0.049 | p = 0.803  r = -0.020 | p = 0.275 | p = 0.827 | p = 0.862 |
| **Social functioning** | p = 0.990  r = -0.001 | p = 0.225  r = -0.098 | p = 0.118 | p = 0.344 | p = 0.353 |
| **Pain** | p = 0.034  r = -0.169 | p = 0.129  r = -0.122 | p = 0.452 | p = 0.547 | p = 0.059 |
| **General health** | p = 0.014  r = -0.195 | p = 0.399  r = -0.068 | p = 0.619 | p = 0.024 | p = 0.580 |

Association between age/BMI and QoL parameters were determined by Pearson's and Spearman's correlation coefficients. Comparisons between two groups (males vs. females, first vs. repeated hospitalization) were analyzed by unpaired t tests or Mann–Whitney tests. Comparisons between three groups (education levels) were assessed by one-way ANOVAs or Kruskal–Wallis tests.
