# Supplementary material for: Physical activity habits and their effects on quality of life in patients with addiction: data from the Czech Republic
Source: Curr Psychol. 2023 Mar 28:1–8. Online ahead of print. doi: 10.1007/s12144-023-04402-w (PMC10043521; doi:10.1007/s12144-023-04402-w)
Supplement: Supplementary file 3 — Supplementary Material 3 [file 12144_2023_4402_MOESM3_ESM.docx]

**Figure Legends**

**Fig. 1.** **(A)** Comparison of quality of life in the patients who reported RPA before hospitalization (Groups A and B) with physically inactive patients (Groups C and D). **(B)** Comparison of quality of life in the patients who engaged in RPA during hospitalization (Groups A and C) with that in physically inactive patients (Groups B and D). Data are presented as the mean ± standard deviation.

**Fig. 2.** **(A)** Comparison of quality of life in the patients who engaged in RPA before and during hospitalization (Group A) with patients who stopped RPA after hospitalization (Group B). **(B)** Comparison of quality of life in the patients who did not engage in RPA before and during hospitalization (Group D) with a group who changed habits and initiated RPA after hospitalization (Group C). Data are presented as the mean ± standard deviation.
